# Efficacy and Safety of Cemiplimab for the Management of Non-Melanoma Skin Cancer: A Drug Safety Evaluation

**DOI:** 10.3390/cancers16091732

**Published:** 2024-04-29

**Authors:** Luca Potestio, Massimiliano Scalvenzi, Aimilios Lallas, Fabrizio Martora, Luigi Guerriero, Luigi Fornaro, Laura Marano, Alessia Villani

**Affiliations:** 1 Section of Dermatology, Department of Clinical Medicine and Surgery, University of Naples Federico II, 80138 Naples, Italy; 2 First Department of Dermatology, School of Medicine, Faculty of Health Sciences, Aristotle University, 541 24 Thessaloniki, Greece

**Keywords:** cemiplimab, basal cell carcinoma, squamous cell carcinoma, systemic treatments, skin cancer

## Abstract

**Simple Summary:**

Basal cell carcinoma (BCC) and cutaneous squamous cell carcinoma (cSCC) are the commonest types of non-melanoma skin cancer. Surgical excision is the mainstay of treatment for both tumors. However, tumor features and patients’ comorbidities may limit the use of these techniques, making the treatment challenging. The introduction of oral therapies targeting a pathogenetic pathway in BCC has revolutionized the therapeutic scenario. However, there are still patients unresponsive or intolerant to these drugs. In this context, cemiplimab has been approved as second-line treatment. As regards SCC, cemiplimab was the first systemic therapy approved. The objective of this manuscript was to investigate the efficacy and safety of cemiplimab for the management of BCC and cSCC. Cemiplimab has a durable and significant effect for the management of BCC and CSCC, with a favorable safety profile.

**Abstract:**

Non-melanoma skin cancer includes several types of cutaneous tumors, with basal cell carcinoma (BCC) and cutaneous squamous cell carcinoma (cSCC) as the commonest. Among the available therapeutic options, surgical excision is the mainstay of treatment for both tumors. However, tumor features and patients’ comorbidities may limit the use of these techniques, making the treatment challenging. As regards BCC, even if hedgehog inhibitors revolutionized the therapeutic scenario, there are still patients unresponsive or intolerant to these drugs. In this context, cemiplimab has been approved as second-line treatment. As regards SCC, cemiplimab was the first systemic therapy approved. The objective of this manuscript was to investigate the efficacy and safety of cemiplimab for the management of BCC and cSCC. Cemiplimab has a durable and significant effect for the management of BCC and CSCC, with a favorable safety profile. Different specialists including oncologists, radiologists, dermatologists, and surgeons are required to guarantee an integrated approach, leading to the best management of patients. Moreover, the collaboration among specialists will allow them to best manage the TEAEs, reducing the risk of treatment suspension or discontinuation. Certainly, ongoing studies and more and more emerging real-world evidence, will allow us to better characterize the role of cemiplimab for the management of advanced non-melanoma skin cancer.

## 1. Introduction

Non-melanoma skin cancer encompasses various cutaneous tumors, with basal cell carcinoma (BCC) and cutaneous squamous cell carcinoma (cSCC) being the most prevalent, comprising approximately 70% and 20% of non-melanoma skin cancer cases, respectively [1,2]. Other types, such as Merkel cell carcinoma and dermatofibrosarcoma protuberans, exist but will not be addressed in this manuscript [1,2]. The incidence of BCC and cSCC is on the rise due to factors like population aging and increased ultraviolet radiation exposure [3,4,5]. Risk factors include immunosuppression, chronic inflammation, fair skin, and sun exposure (e.g., military personnel and outdoor workers exposed to excessive solar radiation), among others [3,4,5]. cSCC also has unique risk factors like papilloma virus infection and arsenic exposure [3,4,5]. Of interest, ultraviolet radiation (UVR) induces electrical, chemical, and biological signals to be sent to the brain, endocrine, and immune systems, as well as other central organs, which in concert regulate body homeostasis [6]. In this context, among possible preventive strategies, photo-neuro-immuno-endocrinology can offer novel therapeutic approaches [6].

BCC typically presents as asymptomatic, enlarging, and often bleeding lesions in sun-damaged areas, while cSCC usually manifests as solitary red scaly plaques or nodules in sun-exposed areas [7]. BCC metastases are rare, while cSCC can metastasize in 3–7% of cases [7]. Surgical excision is the primary treatment for both, though Mohs micrographic surgery and various destructive therapies like topical drugs, radiation therapy, and cryotherapy are also used based on tumor and patient factors [7,8,9,10,11,12,13].

For BCC, understanding its pathogenesis, particularly the role of hedgehog (HH) signaling, led to hedgehog pathway inhibitors (HHIs) like sonidegib and vismodegib, which have shown effectiveness [7,8,9,10,11,12,13]. However, some patients do not respond well or tolerate HHIs, necessitating alternative treatments [7,8,9,10,11,12]. Similarly, despite the fact that cSCC are commonly treated with topical therapies or surgery, new systemic therapies are needed for metastatic (mcSCC) and locally advanced (lacSCC) [13,14].

In this context, cemiplimab, a fully human monoclonal antibody targeting programmed cell death-1 (PD-1), has recently been approved for both diseases [14,15,16]. This review of clinical trials aims to discuss cemiplimab’s efficacy and safety in managing BCC and cSCC.

## 2. Material and Methods

A review of the current literature was carried out by searching the following terms, “cemiplimab”, “basal cell carcinoma”, “cutaneous squamous cell carcinoma”, “trial”, “adverse events”, “efficacy”, and “safety” on the PubMed, Google Scholar, Embase, Cochrane Skin, and clinicaltrials.gov databases (21 January 2024). In particular, reviews, meta- analyses, and clinical trials were considered. On the contrary, real-life studies, case reports, and case series were excluded, not fitting with the aim of the manuscript. Non-English language manuscripts and articles regarding other types of cancers were excluded. References were also reviewed to include articles that could have been missed. Then, the research was refined by reviewing the abstracts and texts of collected articles. This article is based on previously conducted studies and does not contain any studies with human participants or animals performed by any of the authors.

## 3. Introduction to Cemiplimab

Cemiplimab, a fully human monoclonal antibody of the IgG4 class, binds to the programmed cell death-1 (PD-1) receptor, inhibiting its interaction with PD-L1 and PD-L2 ligands. This mechanism enhances anti-tumoral T-cell responses [17]. It has regulatory approval for treating adult patients with mCSCC or laCSCC who are ineligible for curative surgery or radiation, and for adult patients with locally advanced BCC (laBCC) or metastatic BCC (mBCC) who have either progressed on or are intolerant to a hedgehog pathway inhibitor. The recommended dosage is 350 mg every 3 weeks (Q3W) via intravenous infusion over 30 min until disease progression or unacceptable toxicity [18]. Additionally, cemiplimab is authorized for managing non-small cell lung cancer and cervical cancer (Table 1) [18].

### 3.1. Pharmacodynamics

Cemiplimab inhibits the interaction between the PD-1 receptor and its ligands, PD-L1 and PD-L2. Ordinarily, this interaction suppresses the T cell function, including proliferation, cytotoxic activity, and cytokine secretion, as PD-L1 and PD-L2 are expressed by antigen-presenting cells and sometimes by tumor cells [18]. By blocking PD-1 from binding to PD-L1 and PD-L2, cemiplimab enhances T cell responses, fostering anti-tumor activity [18].

### 3.2. Pharmacokinetics

Data on the pharmacokinetics of cemiplimab were obtained from 1063 patients with various solid tumors [18]. At the recommended dosage of 350 mg every 3 weeks (Q3W), steady-state mean drug concentrations ranged from a Ctrough of 59 mg/L to a Cmax of 171 mg/L [19,20]. Steady-state exposure is typically achieved after approximately 4 months of treatment [19,20]. Similar steady-state drug exposure is observed in solid tumor patients at both 350 mg Q3W and 3 mg/kg every 2 weeks (Q2W) [19,20]. Cemiplimab, administered intravenously, exhibits complete bioavailability [19,20]. It primarily distributes within the vascular system, with a steady-state volume of distribution of 5.9 L [19,20]. Median Tmax occurs at the conclusion of the 30-minute infusion [19,20]. Given its protein nature, cemiplimab metabolism has not been assessed; however, it is anticipated to degrade into small peptides and individual amino acids [19,20]. Clearance of cemiplimab is linear at doses ranging from 1 to 10 mg/kg Q2W, with an initial clearance after the first dose of approximately 0.25 L/day, decreasing by 11% over time to reach a steady-state clearance of 0.22 L/day, resulting in a dosing interval half-life of 22 days [19,20]. Notably, at doses of 1–10 mg/kg Q2W, cemiplimab pharmacokinetics demonstrate linearity and dose proportionality, indicating saturation of the systemic target-mediated pathway [19,20]. Factors such as gender, age, body weight, race, albumin level, cancer type, and mild to moderate hepatic or renal impairment do not appear to significantly impact the pharmacokinetic properties of cemiplimab [19,20].

### 3.3. Special Population

Regarding the utilization of cemiplimab in specific populations, there is a lack of data concerning its use in patients under 18 years of age [18]. Conversely, no dosage adjustment is deemed necessary for elderly patients [18]. Likewise, dose modification is unnecessary for patients with renal impairment, although studies in individuals with severe hepatic impairment are lacking [18]. Consequently, cemiplimab is deemed suitable for use in patients with mild to moderate hepatic impairment [18].

### 3.4. Drug to Drug Interactions

Research on pharmacokinetic drug interactions involving cemiplimab has not been conducted [18]. Generally, systemic corticosteroids (with the exception of physiological doses, ≤10 mg/day prednisone or equivalent) or immunosuppressants should be avoided before initiating cemiplimab, as they could potentially disrupt its pharmacodynamic activity and efficacy [18]. However, these medications can be administered after the initiation of cemiplimab to manage immune-mediated adverse reactions [18].

## 4. Basal Cell Carcinoma

Cemiplimab has received approval as a standalone therapy for managing adult patients with laBCC or mBCC who have either progressed on or are intolerant to hedgehog pathway inhibitors (HHIs) [18]. The efficacy and safety of cemiplimab were assessed in an open-label, multicenter, phase II study (NCT03132636) involving 84 patients with laBCC and 54 individuals with mBCC who were unresponsive or intolerant to HHI treatment [21]. The study is summarized in Table 2.

Patients received cemiplimab intravenously at a dosage of 350 mg every 3 weeks for up to 93 weeks or until disease progression or unacceptable toxicity occurred [21]. The primary endpoint was the achievement of objective response (OR), defined as the percentage of patients exhibiting the best overall response of complete (CR) or partial response (PR) [21]. The majority of laBCCs were situated at the head and neck site in both groups (75, 89.3%), with lung metastases being the most prevalent (32, 59.3%) [21].

In the laBCC group, an OR was observed in 26 patients (31.0%), with 5 (6.0%) experiencing CR and 21 (38.9%) achieving PR [19]. The median time to response was 4.3 months [21]. Furthermore, the Kaplan–Meier estimated proportion of patients alive and without disease progression was 76% at 6 months and 57% at 12 months [21]. Regarding safety, grade 3–4 treatment-emergent adverse events (TEAEs) were reported in 40 patients (47.6%), with hypertension and colitis being the most common (4, 4.8%), followed by fatigue, urinary tract infection, and visual impairment (3, 3.6%) [21]. Additionally, no grade 4 or grade 5 immune-related adverse events (AEs) were recorded, and no treatment-related deaths occurred [21].

In the mBCC cohort, an OR was reported in 12 patients (22.2%), with 2 (3.7%) achieving CR and 10 (18.5%) experiencing PR (mean time to response: 3.1 months) [22]. The Kaplan–Meier estimated proportion of patients alive and without disease progression was 100% at 6 months and 58% at 12 months [22]. Regarding safety, TEAEs of any grade occurred in 51 patients (94.4%), predominantly fatigue (23, 42.6%), diarrhea (20, 37.0%), constipation (12, 22.2%), and hypertension (12, 22.2%). TEAEs with at least a grade of 3 were reported in 23 patients (42.6%), with only hypertension (6, 11.1%) occurring in more than two subjects [22]. Overall, four patients (7.4%) discontinued treatment due to AEs [22]. Serious TEAEs were observed in sixteen patients (29.6%), and one patient each died from staphylococcal pneumonia and hemoptysis; however, neither death was considered related to cemiplimab [22].

## 5. Cutaneous Squamous Cell Carcinoma

Cemiplimab has been approved as a standalone treatment for adult patients with mcSCC or lacSCC who are not suitable for curative surgery or radiation therapy. Main trials are summarized in Table 3.

The efficacy and safety of cemiplimab in treating mcSCC and lacSCC were investigated in two open-label, non-randomized studies: Study 1423 and Study 1540 (EMPOWER-CSCC 1) [18,23,24]. Study 1423 enrolled 26 patients, while Study 1540 included 193 patients [18,23,24]. Exclusion criteria for both studies were similar and included autoimmune disease requiring systemic immunosuppressant agents within the past 5 years, history of pneumonitis within the last 5 years, solid organ transplant, prior treatment with immune checkpoint inhibitor therapy, active infection requiring therapy, chronic lymphocytic leukemia, brain metastases, or Eastern Cooperative Oncology Group performance score (PS) ≥ 2 [18,23,24].

Patients in Study 1423 received intravenous cemiplimab at a dosage of 3 mg/kg every 2 weeks (Q2W) for up to 48 weeks, while those in Study 1540 received the same dosage for up to 96 weeks. Additionally, a supplementary group in Study 1540 received cemiplimab 350 mg every 3 weeks (Q3W) for up to 54 weeks [18,23,24].

In Study 1423, an objective response rate (ORR) was achieved by half of the patients, with 7.7% and 42.3% experiencing complete response (CR) and partial response (PR), respectively [18,23,24]. The median duration of response (DOR) was not reached, and a disease control rate of 65% with a median time to response of 2.3 months was observed [18,23,24]. Fatigue was the most common adverse event, reported by 27% of patients, followed by decreased appetite, diarrhea, constipation, hypophosphatemia, hypercalcemia, nausea, and urinary tract infection [18,23,24]. Five deaths occurred, three due to disease progression, one due to an unknown cause in a patient who discontinued treatment due to disease progression and was subsequently lost to follow up, and one due to an adverse event [18,23,24].

In phase II Study 1540, 193 patients (83% male, mean age 72 years, range 38–96 years) were divided into three groups: Group 1 comprised patients with metastatic cSCC (mcSCC) receiving cemiplimab 3 mg/kg every 2 weeks (Q2W) (59 patients), Group 2 included patients with lacSCC receiving cemiplimab 3 mg/kg Q2W (78 patients), and Group 3 consisted of patients with mcSCC receiving cemiplimab 350 mg every 3 weeks (Q3W) (56 patients) [18,24]. The primary endpoint was achieving objective response rate (ORR), determined by a composite endpoint integrating independent central review assessments of radiologic data (RECIST 1.1) and digital medical photography. Secondary endpoints comprised duration of response (DOR), progression-free survival (PFS), overall survival (OS), and complete response (CR). Median follow-up durations were 18.5 months for Group 1, 15.5 months for Group 2, and 17.3 months for Group 3 [18,24].

ORR rates were 47.5% and 43.6% for Group 1 and Group 2, respectively. CR rates were 6.8%, and 12.8% for these groups, with 40.7% and 30.8% of PR, respectively [18,24]. Unfortunately, data on Group 3 are not available.

In Group 1, the predominant adverse events included diarrhea (27%), fatigue (24%), nausea (17%), constipation (15%), and rash (15%) [18,24]. Treatment discontinuation due to adverse events occurred in four patients (7%). Grade 3 or higher treatment-emergent adverse events (TEAEs) reported in more than one patient included cellulitis, pneumonitis, hypercalcemia, pleural effusion, and death [18,24]. Overall, 11 deaths were recorded: 8 attributed to disease progression and 3 to adverse events (complications of pneumonia, death during sleep, and duodenal ulcer with esophagitis and hypercalcemia leading to deep-vein thrombosis) [18,24].

In Group 2, 34 patients (44%) experienced at least one grade 3–4 treatment-emergent adverse event (TEAE), with hypertension (n = 6, 8%) and pneumonia (n = 4, 5%) being the most common [18,24]. Treatment discontinuation was necessary for six patients (8%), including one with grade 4 pneumonia and grade 4 pneumonitis, and another with grade 3 hepatitis, increased alanine aminotransferase, elevated aspartate aminotransferase, and increased alkaline phosphatase [18,24]. The remaining four patients discontinued treatment due to grade 4 pneumonitis, grade 3 proctitis, grade 3 encephalitis, and grade 1 arthralgia, respectively [18,24]. Additionally, 23 subjects (29%) experienced serious TEAEs, with 7 (9%) deemed treatment-related, most commonly pneumonitis (n = 3, 4%) [18,24]. Two deaths (3%) were reported, one of which occurred 10 days after the onset of aspiration pneumonia and was considered related to the study treatment [18,24]. Unfortunately, detailed data on Group 3 are not available [18,24].

The efficacy and safety of cemiplimab were evaluated in a pilot phase 2 study, aiming to assess its use as neoadjuvant therapy in patients with resectable stage II, III, or IV (M0) cSCC [23]. A total of 79 subjects received cemiplimab 350 mg Q3W for up to four doses before undergoing surgery with curative intent [25]. The primary endpoint was a pathological complete response (CR), defined as the absence of viable tumor cells in the surgical specimen. Secondary endpoints included a pathological major response (the presence of viable tumor cells constituting ≤10% of the surgical specimen), objective response rate (ORR) on imaging, and adverse events [25].

Results showed a pathological complete response in 40 patients (51%) and a pathological major response in 10 patients (13%). Additionally, an ORR on imaging was observed in 54 patients (68%) [25]. Adverse events were common, with 69 subjects (87%) experiencing at least one adverse event of any grade, most commonly fatigue (30%), diarrhea (14%), nausea (14%), and maculopapular rash (14%) [25]. Among these, 14 patients (18%) experienced a treatment-emergent adverse event with a grade of at least 3 [25].

Lastly, a phase IV, non-interventional study named CASE (CemiplimAb-rwlc Survivorship and Epidemiology) has been planned [26]. This study enrolls patients with advanced cSCC who have recently started or are planning to receive cemiplimab in a real-world setting [26]. The primary objectives of the trial include assessing the effectiveness and safety of cemiplimab at the labeled dosage (350 mg IV, Q3W) in a real-life scenario [26]. Additionally, the study aims to evaluate patient experiences, including quality of life (QoL) and performance status [26].

## 6. Expert Opinion

BCC stands as the most prevalent human cancer. While surgery remains the preferred treatment, systemic therapies become necessary for mBCC and laBCC cases [27]. HHIs have been a game changer, yet some patients remain unresponsive or intolerant, thus necessitating alternative options [28,29]. The approval of cemiplimab has introduced a new avenue, showing efficacy as a second-line option [28,29]. In phase II trials, cemiplimab demonstrated objective responses in 31% and 22.2% of laBCC and mBCC cases, respectively, with differing adverse event profiles compared with HHIs.

Conversely, the treatment landscape for advanced cSCC differs [30]. Although less common than BCC, cSCC poses a higher risk of metastasis with no established first-line systemic treatments [31]. Cemiplimab’s approval marked a milestone, serving as the first systemic therapy for both lacSCC and mcSCC. While treatment duration guidelines are yet to be defined, initial data from EMPOWER-CSCC 1 suggest promising efficacy with an objective response rate ranging from 44.9% to 50.8%. Ongoing studies aim to ascertain long-term effectiveness and identify potential response markers. Regarding safety, cemiplimab’s profile, though acceptable, warrants monitoring for immune-specific adverse reactions.

Notably, cemiplimab’s neoadjuvant use in resectable cSCC demonstrated favorable results, with a notable pathological complete response rate. 

## 7. Conclusions

Overall, cemiplimab demonstrates durable efficacy with a favorable safety profile in managing mBCC, laBCC, lacSCC, and mcSCC. An integrated approach involving oncologists, radiologists, dermatologists, and surgeons is crucial for optimal patient management. Collaboration among specialists can help mitigate treatment-related adverse events, minimizing the risk of treatment interruptions or discontinuations. Ongoing research and real-world evidence will further delineate the role of cemiplimab in the management of advanced non-melanoma skin cancer management. Finally, real-life studies will allow to evaluate the use of cemiplimab also in patients which are often excluded from clinical trials such as transplanted patients or patient with auto-immune diseases.

## Figures and Tables

**Table 1:** Current therapeutic approval of cemiplimab.

Metastatic or locally advanced cutaneous squamous cell carcinoma which are not candidates for curative surgery or curative radiation.
Locally advanced or metastatic basal cell carcinoma which have progressed on or are intolerant to a hedgehog pathway inhibitor.
Adult patients with non-small cell lung cancer (NSCLC) expressing PD-L1 (in ≥50% tumor cells), with no EGFR, ALK, or ROS1 aberrations, who have locally advanced NSCLC, who are not candidates for definitive chemoradiation, or metastatic NSCLC.
In combination with platinum-based chemotherapy is indicated for the first-line treatment of adult patients with NSCLC expressing PD-L1 (in ≥1% of tumor cells), with no EGFR, ALK, or ROS1 aberrations, who have locally advanced NSCLC, who are not candidates for definitive chemoradiation, or metastatic NSCLC.
Adult patients with recurrent or metastatic cervical cancer and disease progression on or after platinum-based chemotherapy.

**Table 2:** Clinical trials investigating the efficacy and safety of cemiplimab in BCC management.

Trial	Phase	Patients	Treatment	Results	Safety
NCT03132636	II	138: - laBCC: 84 - mBCC: 54	cemiplimab 350 mg intravenously Q3W for up to 93 weeks or until progression or unacceptable toxicity	laBCC: - OR: 26 (31.0%); - CR: 5 (6.0%): - PR: 21 (38.9%). mBCC: - OR: 12 (22,2%); - CR: 2 (3.7%); - PR: 10 (18.5%).	laBCC: TEAEs of any grade occurred in 82 (97.6%), with fatigue (25, 29.8%), diarrhea (20, 23.8%), and pruritus (18, 21.4%) as the commonest. mBCC: TEAEs of any grade occurred in 51 patients (94.4%), predominantly fatigue (23, 42.6%), diarrhea (20, 37.0%), constipation (12, 22.2%), and hypertension (12, 22.2%).

Q3W: every 3 weeks; OR: objective response; CR: complete response; PR: partial response; TEAE: treatment-emergent adverse events.

**Table 3:** Main clinical trials investigating the efficacy and safety of cemiplimab in cSCC management.

Trial	Phase	Patients	Treatment	Results	Safety
Study 1423	I	26: - mcSCC: 16 - lacSCC: 10	3 mg/kg Q2W up to 40 weeks	OR: 13 (50.0%) CR: 0 (0%) PR: 13 (50.0%)	TEAEs of any grade occurred in 26 (100%) patients, with fatigue (7, 26.9%), constipation (4, 15.4%), decreased appetite (4, 15.4%), diarrhea 4 (15.4%), hypercalcemia (4, 15.4%), hypophosphatemia (4, 15.4%), nausea (4, 15.4%), urinary tract infection (4, 15.4%) as the commonest.
Study 1540	II	193: - mcSCC: 59 (Group 1) - lacSCC: 78 (Group 2) - mCSCC: 56 (Group 3)	Group 1: 3 mg/kg Q2W up to 96 weeks; Group 2: 3 mg/kg Q2W up to 96 weeks; Group 3: 350 Q2W up to 96 weeks.	Group 1: - OR: 28 (47.5%); - CR: 4 (6.8%); - PR: 24 (40.7%). Group 2: - OR: 34 (43.6%); - CR: 10 (12.8%); - PR: 24 (30.8%). Group 3: no data.	Group 1: TEAEs of any grade occurred in 59 (100%) patients, with diarrhea (16, 27.1%), fatigue (14, 23.7%), and nausea (10, 16.9%) as the commonest. Group 2: TEAEs of any grade occurred in 78 (100%) patients, with fatigue (33, 42.3%), diarrhea (21, 26.9%), and pruritus (21, 26.9%) as the commonest. Group 3: no data.

Q2W: every 2 weeks; OR: objective response; CR: complete response; PR: partial response; na: no data; TEAE: treatment-emergent adverse events.

## Data Availability

The authors confirm that the data supporting the findings of this study are available within the article.
